# Cardiac myxoma misdiagnosed as infective endocarditis: a case of Carney complex

**DOI:** 10.1186/s13019-020-01238-4

**Published:** 2020-07-25

**Authors:** Chang Hun Kim, Hyung Gon Je, Min Ho Ju, Chee-Hoon Lee

**Affiliations:** Department of Thoracic and Cardiovascular Surgery, Pusan National University Yangsan Hospital, Medical Research Institute of Pusan National University, Geumo-ro 20, Beomeo-ri, Mulgeum-eup, Yangsan-si, Gyeongsangnam-do 50612 Republic of Korea

**Keywords:** Myxoma, Infective endocarditis, Cerebral infarction, Cushing syndrome, Carney complex

## Abstract

**Background:**

Infective endocarditis and cardiac myxoma have common features including fever, systemic embolism and intra-cardiac masses. For this reason, these diseases are often misdiagnosed one for another despite proper imaging studies. Herein, we report a case of suspected infective endocarditis in a patient with acute stroke, fever and a mass adjacent to the mitral valve.

**Case presentation:**

A 24-year-old male patient presented with recurrent fever and stroke. In view of a history of Cushing syndrome and a mobile mass in the left atrium, infective endocarditis was highly suspected. He was transferred for emergency cardiac surgical intervention. During surgery, intraoperative transesophageal echocardiography revealed a 7 cm mass attached to the interatrial septum. The mass was excised through right mini-thoracotomy and pathological examination confirmed the presence of a myxoma. Based on the above clinical findings and genetic analysis, the diagnosis of Carney complex was confirmed.

**Conclusions:**

Infective endocarditis and cardiac myxoma have common features and can be misdiagnosed. If a young patient presenting with embolic stroke had a history of an endocrine neoplasm, Carney complex should be considered in the differential diagnosis of infective endocarditis.

## Background

At least 20% of all ischemic strokes originate in the heart. Especially, young patients presenting with acute stroke should be evaluated for an intra-cardiac source of embolism. Among them, infective endocarditis (IE) and cardiac myxoma (CM) share common features such as fever, systemic embolism, and an intra-cardiac mass. A recent publication also showed a case of CM initially misdiagnosed as IE [1]. These diseases can be easily diagnosed using imaging studies, but there is a possibility of misdiagnosis. We report a case of suspected IE in a patient with recurrent fever, acute stroke, and a mass adjacent to the mitral valve.

## Case presentation

**Fig. 1:**
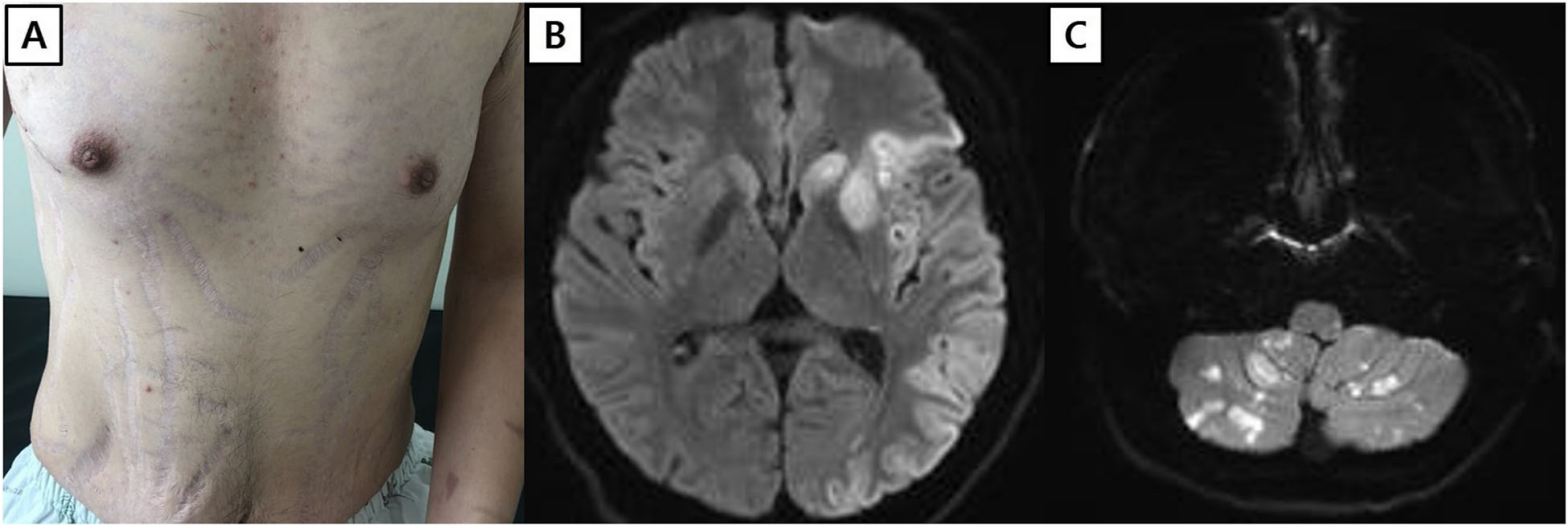
The torso of the patient shows skin striae due to Cushing’s syndrome (**a**). Magnetic resonance images show acute infarction on the area of left middle cerebral artery (**b**) and both cerebellum (**c**)

A 24-year old male patient visited the emergency department of a tertiary hospital for increased drowsiness and fever. The patient had a history of Cushing’s syndrome (Fig. 1a) and had been on long-term steroid replacement therapy after resection of a functioning adrenal adenoma 4 years ago. Multiple cerebral infarcts were revealed on brain magnetic resonance imaging (Fig. 1b, c). He was treated with intravenous tissue plasminogen activator (tPA) and mechanical thrombectomy of the left middle cerebral artery. To identify the source of the cerebral embolism, transthoracic echocardiography (TTE) was performed and a large, 4 cm sized, highly mobile mass adjacent to the mitral valve was revealed. After cerebral intervention, the patient presented recurrent fever and hypotension. Based on the patient’s grossly Cushing’s features, long history of steroid usage, fever and hypotension, the cardiologist in the referral hospital highly suspected an IE. For treatment with minimally invasive cardiac surgery, they transferred the patient to our hospital. Upon arrival, he experienced drowsiness and aphasia and his Glasgow Coma Scale (GCS) was recorded at 12/15 (eyes 3, verbal 4, motor 5). The patient presented stable vital signs except mild fever of 37.1 °C. Lab results showed neutrophil-dominant leukocytosis and elevated C-reactive protein (CRP) of 15.19. On account of the potential systemic effects of tPA and risk of hemorrhagic transformation in the area of cerebral infarction, surgery was deferred for 48 h. He was managed with intravenous antibiotics. For further evaluation, we tried transesophageal echocardiography, but the patient’s unclear mentality interfered with the test.

**Fig. 2:**
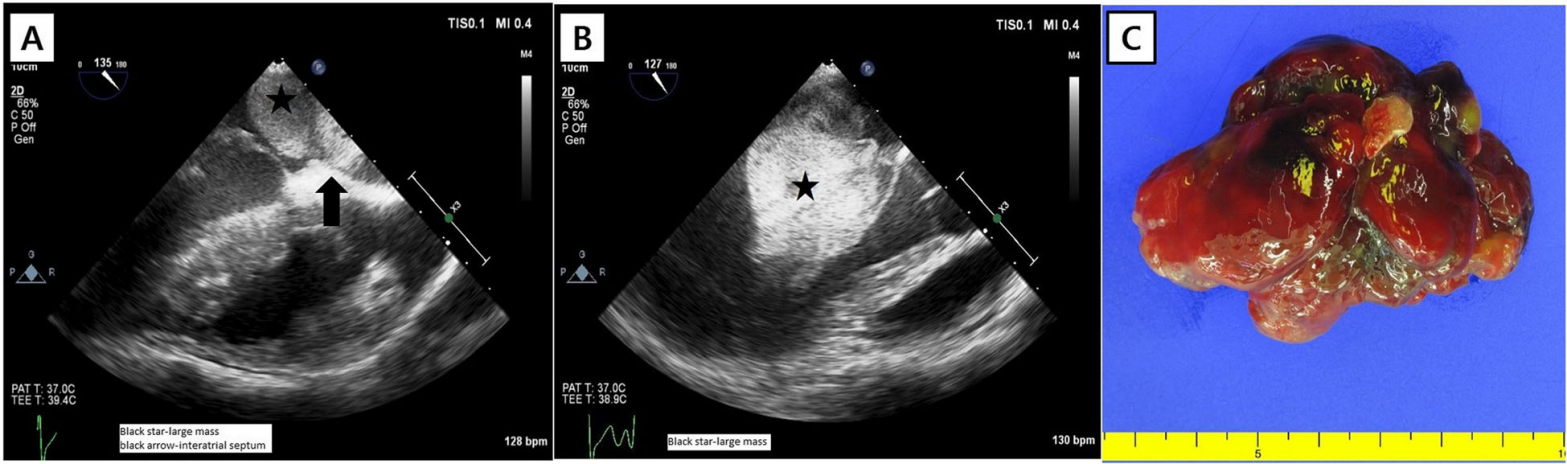
Transesophageal echocardiography shows a large mass (black star) attached to the interatrial septum (black arrow) (**a**), which protrudes into the mitral valve (**b**). The gross specimen of the excised mass is shown (**c**)

During surgery, intraoperative transesophageal echocardiography (TEE) revealed that the mass was not attached to the mitral valve but to the interatrial septum (Fig. 2a). The mass was found obstructing the mitral inflow, leading to functional mitral stenosis (Fig. 2b). We considered the possibility that the mass might be a tumor (possibly CM) rather than a vegetation due to IE. A 6-cm right mini-thoracotomy was performed along the fourth intercostal space, and a cannula was inserted through the right femoral artery and vein (17 Fr and 23 Fr, Edwards Lifescience, Irvine, CA, USA). After aortic cross-clamping with the Glauber clamp (Cardiomedical GmbH, Langenhagen, Germany), cardioplegic arrest was induced with antegrade cold blood cardioplegia. Through the left atriotomy, complete excision of the mass with its stalk and adjacent endocardium was achieved. The mass was 7 cm in size, friable, and multi-lobulated (Fig. 2c). Cardiopulmonary bypass and aortic cross-clamping took 35 and 17 min, respectively. After surgery, mitral valve stenosis or regurgitation was not observed. The patient was extubated 6 h postoperatively and transferred to the ward the next day. Postoperative TTE showed no remnant mass in the left atrium and an intact interatrial septum. The drowsiness and aphasia gradually improved. He was transferred to the Department of Neurology for further management of his neurological sequelae. Pathologic examination confirmed the diagnosis of a myxoma. Carney complex (CNC) was suspected because of the history of endocrine neoplasm and CM. Genetic analysis confirmed the presence of a mutation in the *PPKAR1A* gene. Therefore, he was finally diagnosed with CNC presenting with CM and systemic embolism.

## Discussion and conclusions

In the present case, symptoms and signs such as skin striae in the torso, relapsing fever, a highly mobile mass on the mitral valve, and embolic cerebral infarction led to a high level of suspicion for IE. Additionally, the immunocompromised state due to long-term steroid replacement therapy supported the probability of IE. However, intraoperative TEE helped confirm that the mass was attached to the interatrial septum and not to the mitral valve, thereby showing the mass to be a myxoma. CM is the most frequently occurring benign primary cardiac tumor, accounting for 0.3% of open-heart surgeries [1]. While vegetation in IE is known to cause systemic embolism, cardiac myxoma can also be an important cause of stroke. CM is responsible for 0.5% of all strokes and 10–30% of patients with CM were reported to develop cerebral embolic infarction [2]. Although infected CM is a rare condition, it should also be considered in the differential diagnosis of fever and embolism [3]. Therefore, if a patient with cerebral infarction had a cardiac mass, CM as well as IE should be suspected. To make a differential diagnosis, whenever operating on infective lesions, a part of the specimen should be sent to histopathology rather than all of it going to microbiology.

Most cases of CM are benign and sporadic. Familial forms of myxoma also exist and typically occur in young, male patients in unusual locations, and have a tendency to be multifocal [4]. CNC is a rare autosomal dominant syndrome, characterized by pigmented skin lesions, familial CM forms, and endocrine neoplasms of the adrenocortical, thyroid, or testicular systems [5]. As in the present case, if a patient with CM has a history of endocrine tumors, CNC should be suspected. Surgical resection should be done promptly because of the high risk of complications due to tumoral effects or systemic embolism [6]. Because CM in CNC has a high risk of systemic embolism and recurrence, extensive resection including the stalk with adjacent endocardium is advised and periodic TTE during long-term follow up is mandatory [7, 8] genetic counseling and education for. Finally, genetic counseling and education for close relatives is also important for the treatment of patients with CNC.

In the present case, the precipitous treatment with intravenous tPA and mechanical thrombectomy delayed surgery. For young patients presenting with stroke, prompt evaluation of the source of embolism should be done prior to stroke treatment. Echocardiography and computed tomography can provide valuable information to help establish a treatment plan and minimize the possibility of additional embolic events.

In summary, infective endocarditis might be suspected in patients with a history of recurrent fever, stroke, and a mobile mass in the left atrium. However, due to similarities in symptoms and signs, Carney complex associated cardiac myxoma should also be included in the differential diagnosis, especially in case of young patients with a history of endocrine neoplasms.

## Data Availability

Not applicable.

## Abbreviations

IE: Infective endocarditis
CM: Cardiac myxoma
tPA: Tissue plasminogen activator
TTE: Trans-thoracic echocardiogram
TEE: Transesophageal echocardiography
CNC: Carney complex
